# Supplementary material for: Structural characterization of the YbbAP-TesA ABC transporter identifies it as a lipid hydrolase complex that extracts hydrophobic compounds from the bacterial inner membrane
Source: PLoS Biol. 2025 Nov 25;23(11):e3003427. doi: 10.1371/journal.pbio.3003427 (PMC12646458; doi:10.1371/journal.pbio.3003427)
Supplement: S1 Table — (DOCX) [file pbio.3003427.s001.docx]

**Table S1: Co-evolving residues predicted between YbbA, YbbP and TesA.**

**Top co-evolving residues identified between YbbP and TesA**

| **Rank** | **YbbP** | **TesA** | **CCMPRED Raw score** | **Z-score** | †**Distance (Å)** |
| --- | --- | --- | --- | --- | --- |
| 1 | Asp148 | Arg141 | 0.5 | **34.72** | 6.4 |
| 2 | Asp145 | Arg142 | 0.27 | **17.02** | 10.2 |
| 3 | Glu500 | Ala78 | 0.24 | **14.25** | 9.9 |
| 4 | Gln188 | Ala137 | 0.22 | **12.77** | 6.6 |
| 5 | Thr657 | Gln74 | 0.2 | **11.62** | 7.8 |
| 6 | Ala498 | Ala78 | 0.2 | **11.01** | 8.9 |
| 7 | Gln598 | Gln86 | 0.19 | **10.55** | 8.9 |
| 8 | Gly630 | Lys85 | 0.19 | **10.16** | 12.8 |
| 9 | Ala586 | Asp159 | 0.18 | 9.82 | 53.7 |
| 10 | Thr499 | Ala78 | 0.18 | **9.37** | 9.6 |
| 11 | Asp148 | Glu145 | 0.17 | **8.66** | 11.9 |
| 12 | Pro632 | Ile68 | 0.16 | **8.46** | 10.4 |
| 13 | Phe89 | Arg142 | 0.16 | **8.15** | 9.2 |
| 14 | Thr657 | Phe105 | 0.16 | **8.04** | 7.7 |
| 15 | Gln501 | Gln74 | 0.15 | **7.60** | 10.5 |
| 16 | Thr657 | Gln109 | 0.15 | **7.39** | 9.6 |
| 17 | Gln631 | Ala82 | 0.14 | **6.69** | 8.6 |

**Top coevolving residues identified between YbbP and YbbA**

| **Rank** | **YbbP** | **YbbA** | **CCMPRED Raw score** | **Z-score** | †**Distance (Å)** |
| --- | --- | --- | --- | --- | --- |
| 1 | Gly283 | Lys84 | 0.24 | **15.25** | 7.3 |
| 2 | Gly720 | Lys84 | 0.17 | **9.65** | 6.9 |
| 3 | Thr279 | Phe92 | 0.17 | **9.59** | 8.2 |
| 4 | Leu273 | Ile100 | 0.17 | **9.24** | 7.1 |
| 5 | Gln709 | Met98 | 0.16 | **8.97** | 9.1 |
| 6 | Leu368 | Ser50 | 0.16 | **8.68** | 12.8 |
| 7 | Phe243 | Ser47 | 0.15 | 7.43 | 66.2 |
| 8 | Leu607 | His10 | 0.13 | 6.59 | 88.0 |
| 9 | Thr716 | Ala81 | 0.13 | 6.37 | 45.3 |

**Top coevolving residues identified between TesA and YbbA**

| **Rank** | **TesA** | **YbbA** | **CCMPRED Raw score** | **Z-score** | †**Distance (Å)** |
| --- | --- | --- | --- | --- | --- |
| 1 | Gly40 | Asn176 | 0.13 | 5.01 | 105 |
| 2 | Pro89 | Val203 | 0.13 | 4.75 | 127 |
| 3 | Ala188 | Ser50 | 0.12 | 4.65 | 108 |
| 4 | Ser73 | Val155 | 0.12 | 4.44 | 107 |
| 5 | Ala146 | Pro165 | 0.12 | 4.34 | 119 |
| 6 | Leu135 | Lys184 | 0.12 | 4.18 | 123 |
| 7 | Leu84 | Ile201 | 0.12 | 4.11 | 123 |
| 8 | Glu157 | Asp187 | 0.12 | 4.03 | 141 |
| 9 | Trp91 | Ph94 | 0.12 | 3.99 | 119 |
| 10 | Leu130 | Asn162 | 0.12 | 3.97 | 117 |

†Distance is between C⍺ positions in the YbbAP-TesA cryoEM structure.
