# Supplementary material for: Structural characterization of the YbbAP-TesA ABC transporter identifies it as a lipid hydrolase complex that extracts hydrophobic compounds from the bacterial inner membrane
Source: PLoS Biol. 2025 Nov 25;23(11):e3003427. doi: 10.1371/journal.pbio.3003427 (PMC12646458; doi:10.1371/journal.pbio.3003427)
Supplement: S2 Table — (DOCX) [file pbio.3003427.s002.docx]

**Table S2: CryoEM data and model refinement statistics**

|  | **YbbAP** | **YbbAP +ATP** | **YbbAP-TesA +ATP** |
| --- | --- | --- | --- |
| **Data collection and processing** |  |  |  |
| EMDB code | EMD-51291 | EMD-51292 | EMD-51293 |
| Magnification | 105,000 | 105,000 | 105,000 |
| Voltage (kV) | 300 | 300 | 300 |
| Electron exposure (e^–^/Å^2^) | 50 | 50 | 50 |
| Defocus range (μm) | 800-2,300 | 800-2,300 | 800-2,300 |
| Pixel size (Å) | 0.835 | 0.835 | 0.835 |
| Final number of particles | 94,477 | 52,834 | 29,958 |
| Map Resolution (FSC 0.143) | 4.05 | 3.66 | 4.55 |
| **Model refinement** |  |  |  |
| PDB code | 9GE6 | 9GE7 | 9GE8 |
| Resolution (Å) | 4.05 | 3.66 | 4.55 |
| Sharpened Map B |  |  |  |
| Composition | YbbP, YbbA, YbbA | YbbP, YbbA, YbbA | YbbP, YbbA, YbbA, TesA |
| Protein Residues | 799, 224, 224 | 804, 226, 226 | 804, 225, 225, 172 |
| Ligands | - | 2 ANP, 2 Mg^2+^ | 2 ANP, 2 Mg^2+^ |
| B factors |  |  |  |
| Protein | 202, 190, 185 | 168, 138, 131 | 232, 251, 251, 347 |
| Ligands | - | 126, 117, 120, 96 | 237, 237, 209, 228 |
| Rms Deviations |  |  |  |
| RmsBonds | 0.004 | 0.005 | 0.002 |
| RmsAngles | 0.653 | 0.642 | 0.458 |
| **Validation** |  |  |  |
| Map CC (Proteins) | 0.77, 0.84, 0.84 | 0.83, 0.87, 0.87 | 0.79, 0.86, 0.82, 0.82 |
| Map CC (Ligands) | - | 0.94, 0.95, 0.95, 0.97 | 0.89, 0.89, 0.93, 0.95 |
| Q-score | 0.26, 0.31, 0.29 | 0.41, 0.46, 0.46 | 0.26, 0.27, 0.24, 0.19 |
| Clash-score | 19.98 | 13.4 | 10.28 |
| MolProbity score | 2.04 | 2.33 | 1.75 |
| Ramachandran outliers (%) | 0 | 0 | 0 |
