# Supplementary material for: Structural characterization of the YbbAP-TesA ABC transporter identifies it as a lipid hydrolase complex that extracts hydrophobic compounds from the bacterial inner membrane
Source: PLoS Biol. 2025 Nov 25;23(11):e3003427. doi: 10.1371/journal.pbio.3003427 (PMC12646458; doi:10.1371/journal.pbio.3003427)
Supplement: S3 Table — (DOCX) [file pbio.3003427.s003.docx]

**Table S3: Antibiotic susceptibility testing for TesA and YbbP knockout strains.**

| ***E. coli***  **Strain** | **Plasmid** | **Antibiotic**  **tested** | **Notes** | **MIC Values (μg/mL)** | **Median MIC**  **(μg/mL)** |
| --- | --- | --- | --- | --- | --- |
|  |  |  |  |  |  |
| BW25113 | - | Penicillin G | - | 32, 32, 32, 32 | 32 |
| *Δtesa* | - | Penicillin G | - | 32, 32 | 32 |
| *Δybbp* | - | Penicillin G | - | 32, 32, 32, 16 | 32 |
|  |  |  |  |  |  |
| BW25113 | - | Cephalexin | - | 16, 16, 16, 32 | 16 |
| *Δtesa* | - | Cephalexin | - | 16, 16 | 16 |
| *Δybbp* | - | Cephalexin | - | 16, 16, 16, 16 | 16 |
|  |  |  |  |  |  |
| BW25113 | - | Cephalothin | - | 8, 16 | 12 |
| *Δtesa* | - | Cephalothin | - | 16, 16 | 16 |
| *Δybbp* | - | Cephalothin | - | 16, 16 | 16 |
|  |  |  |  |  |  |
| C43(DE3) | pET28/Empty | Ampicillin | +IPTG +Kan | 8, 8, 8, 8 | 8 |
| C43(DE3) | pET28/YbbAPTesA | Ampicillin | +IPTG +Kan | 8, 8, 8, 8 | 8 |
| C43(DE3) | pET28/YbbAPTesA(S36A) | Ampicillin | +IPTG +Kan | 4, 4, 4, 8 | 4 |
| C43(DE3) | pET28/YbbAP | Ampicillin | +IPTG +Kan | 4, 4, 8, 8 | 6 |
|  |  |  |  |  |  |
| C43(DE3) | pET28/Empty | Cephalexin | +IPTG +Kan | 8, 8, 8, 8 | 8 |
| C43(DE3) | pET28/YbbAPTesA(WT) | Cephalexin | +IPTG +Kan | 8, 8, 16, 16 | 12 |
| C43(DE3) | pET28/YbbAPTesA(S36A) | Cephalexin | +IPTG +Kan | 8, 16, 16, 16 | 16 |
| C43(DE3) | pET28/YbbAP | Cephalexin | +IPTG +Kan | 8, 8, 8, 8 | 8 |
|  |  |  |  |  |  |

All Minimum Inhibitory Concentrations (MICs) measured at 37 °C after 18 h – except C43(DE3) experiments which were measured after 48 h at 37 °C.
