# Supplementary figures and images for: Structural characterization of the YbbAP-TesA ABC transporter identifies it as a lipid hydrolase complex that extracts hydrophobic compounds from the bacterial inner membrane
Source: PLoS Biol. 2025 Nov 25;23(11):e3003427. doi: 10.1371/journal.pbio.3003427 (PMC12646458; doi:10.1371/journal.pbio.3003427)

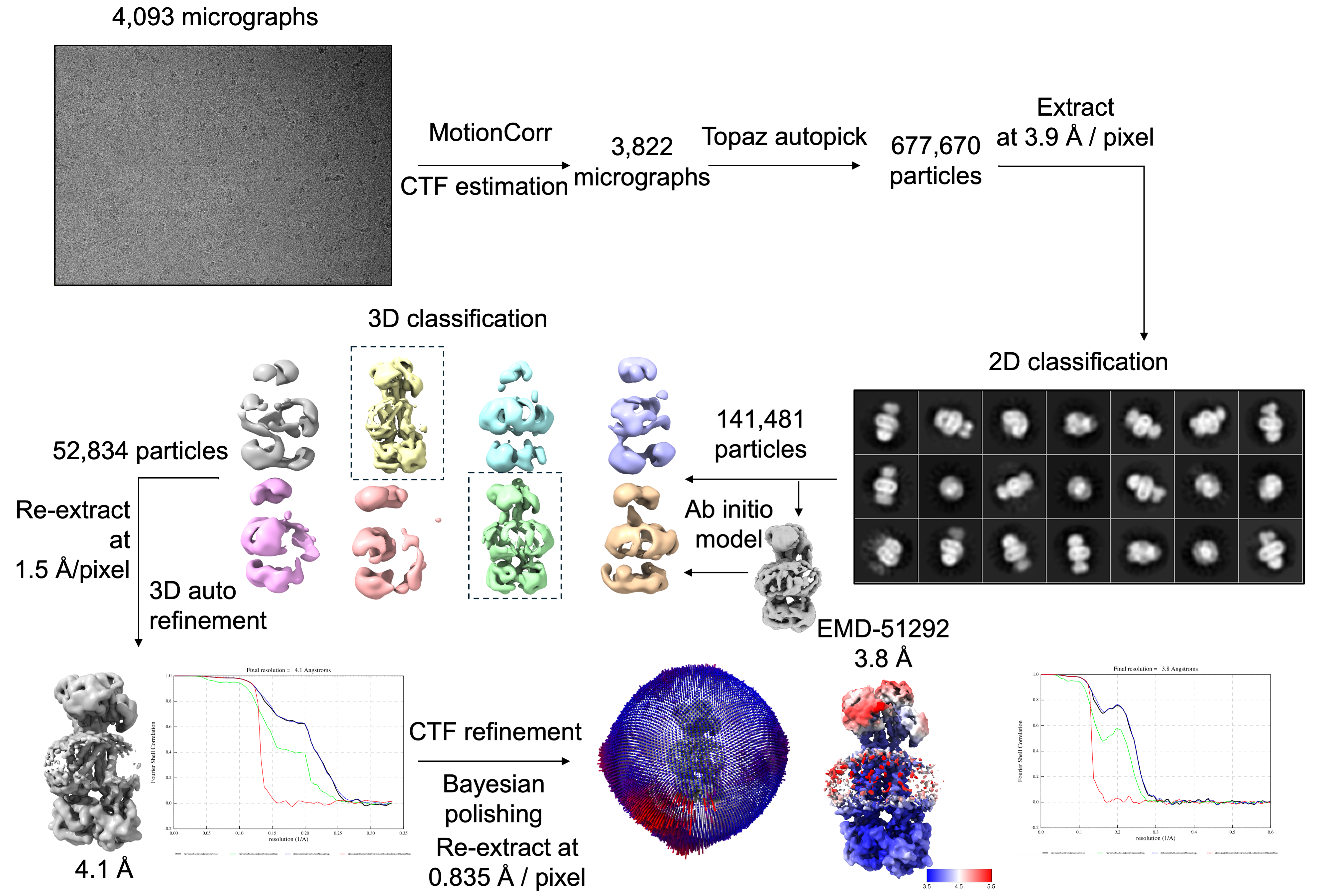

Supplement: S1 Fig — All processing was performed using Relion, with intermediary maps being shown as representatives of multiple jobs. 2D and 3D classifications were performed exhaustively to remove bad particles. The structure is deposited in the Protein Data Bank with accession code 9GE7 with cryoEM maps (sharpened and unsharpened) available from the Electron Microscopy Data Bank under code EMD-51292. (TIFF) [file pbio.3003427.s004.tiff]

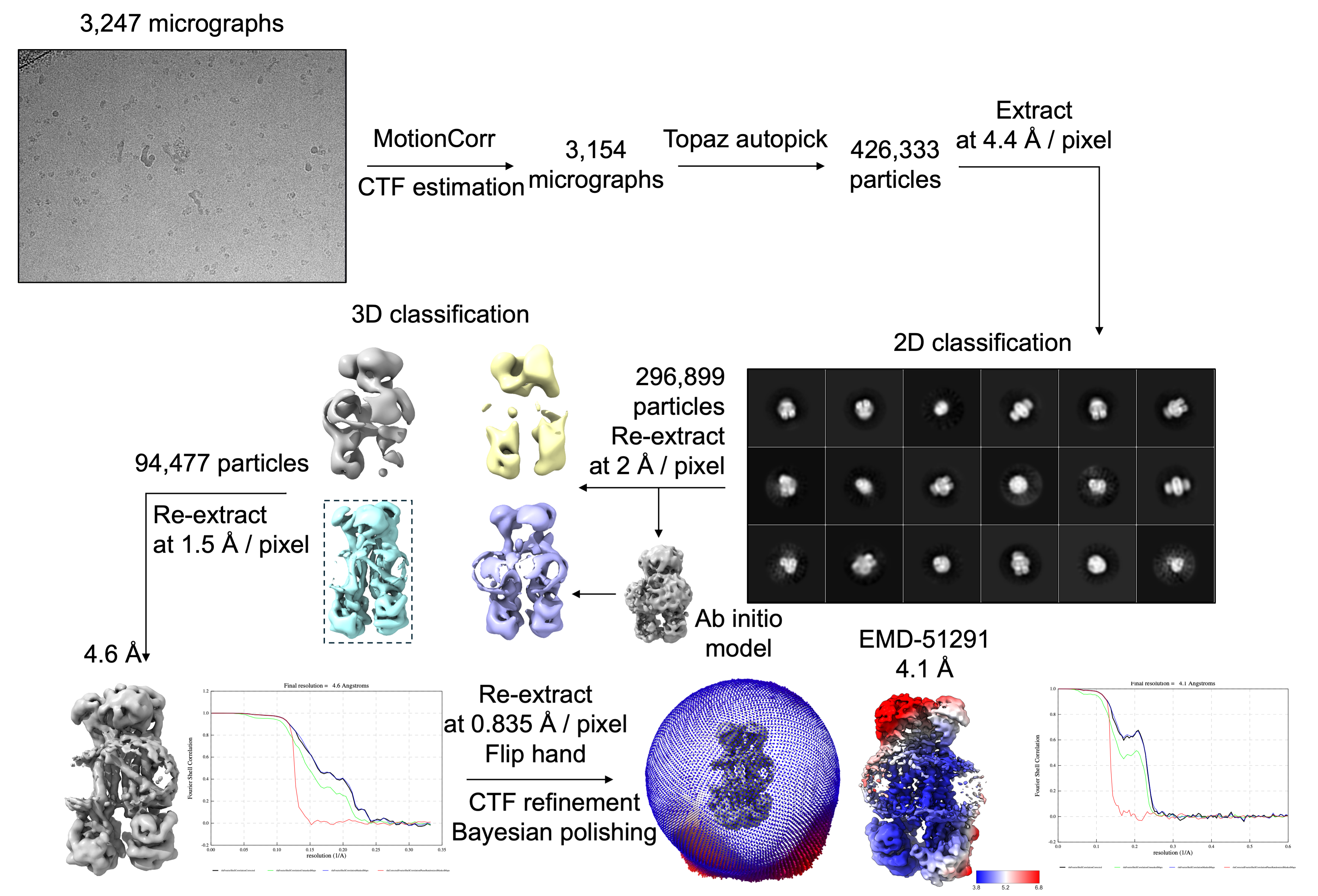

Supplement: S2 Fig — The structure is deposited in the protein data bank with accession code 9GE6. The final maps (sharpened and unsharpened) are available in the EMDB under code EMDB-51291. (TIFF) [file pbio.3003427.s005.tiff]

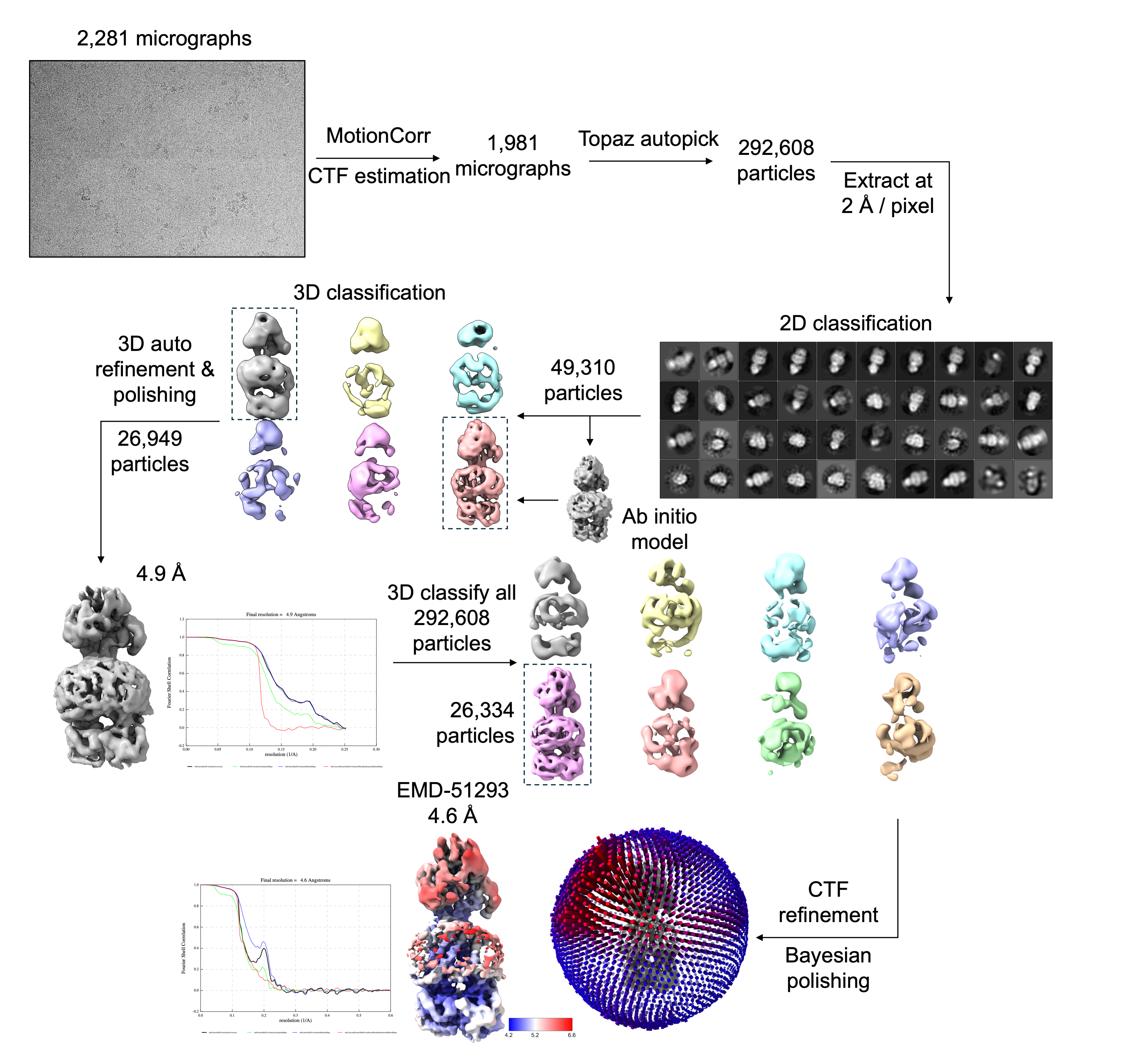

Supplement: S3 Fig — The structure is deposited in the protein data bank with accession code 9GE8. The cryoEM maps (sharpened and unsharpened) are available in the EMDB under code EMD-51293. (TIFF) [file pbio.3003427.s006.tiff]

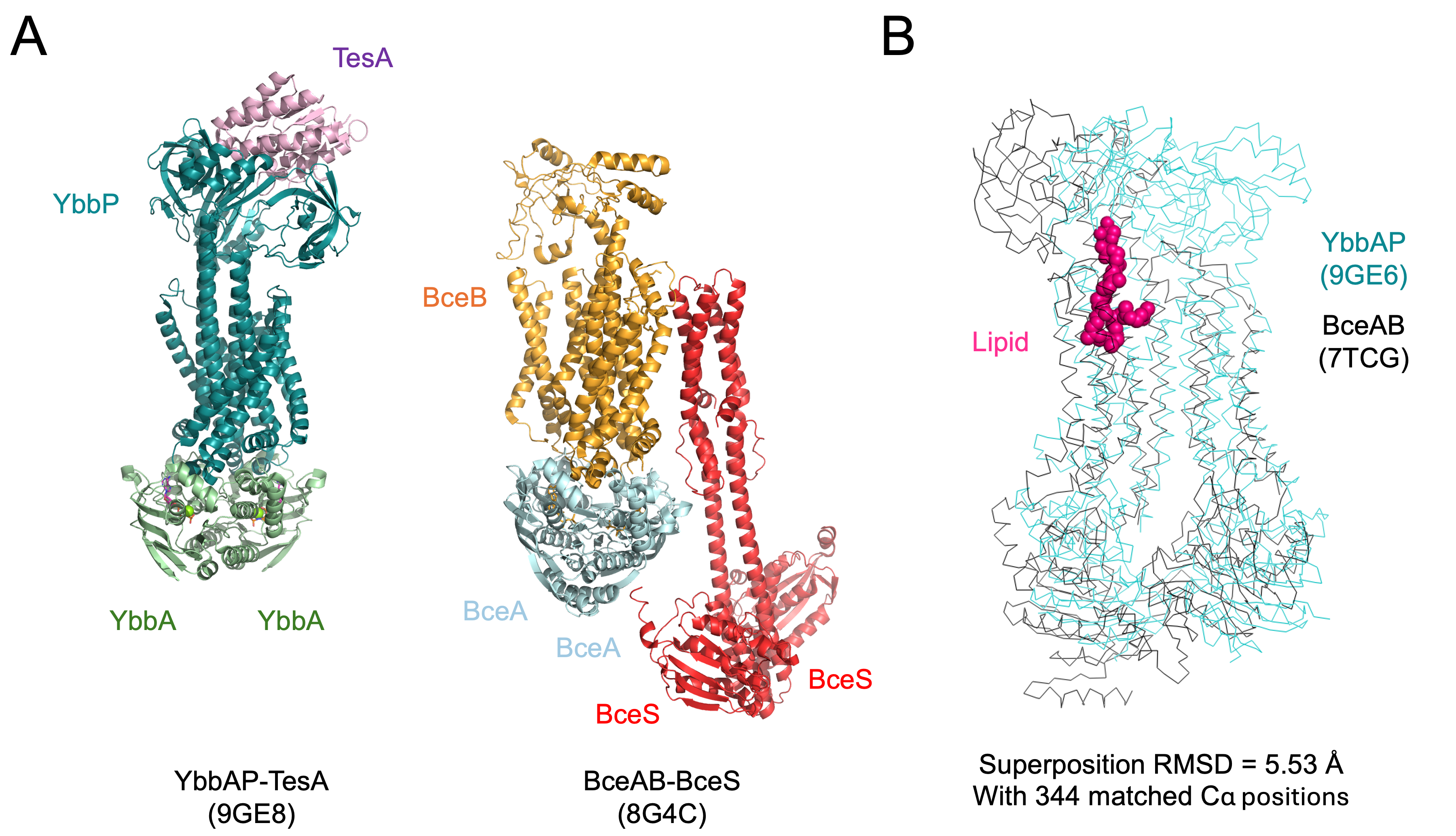

Supplement: S4 Fig — (A) Side-by-side views of YbbAP-TesA and BceAB-BceS. YbbP and BceB have similar 12-helix transmembrane topologies but assemble with distinctive partner proteins. Note also that BceB also has only a single large periplasmic domain, while YbbP has two. (B) Superposition of nucleotide-free YbbAP and nucleotide-free BceAB. A lipid bound in the proposed BceAB UPP-binding pocket is shown in pink and spatially overlaps the proposed periplasmic-side pocket noted in YbbAP. Coordinates for YbbAP-TesA are available from the Protein Data Bank (accession code: 9GE8). Coordinates for BceAB-BceS and BceAB used in comparisons were obtained from the Protein Data Bank (entries 8G4C and 7TCG). (TIFF) [file pbio.3003427.s007.tiff]

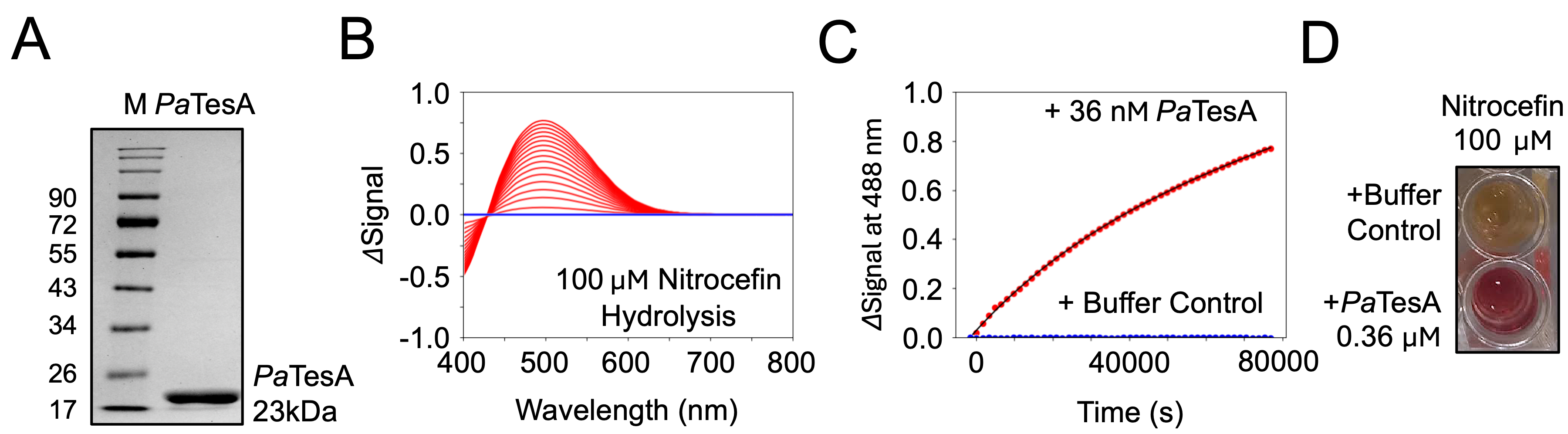

Supplement: S5 Fig — (A) Purified P. aeruginosa TesA with N-terminal tag (PaTesA). (B) Representative example of nitrocefin hydrolysis by PaTesA. Red nested curves represent a series of spectral measurements taken over time. Blue curves indicate the control, where the enzyme was substituted for protein-free buffer. (C) Plots of the change in absorption at 488 nm for enzyme-catalyzed reaction (red dots) and the control (blue dots). A fit from a single exponential function is shown in black. (D) Photograph of two wells after ~18h showing the color change associated with nitrocefin hydrolysis. Underlying data is provided in S11 Data–S13 Data. (TIFF) [file pbio.3003427.s008.tiff]

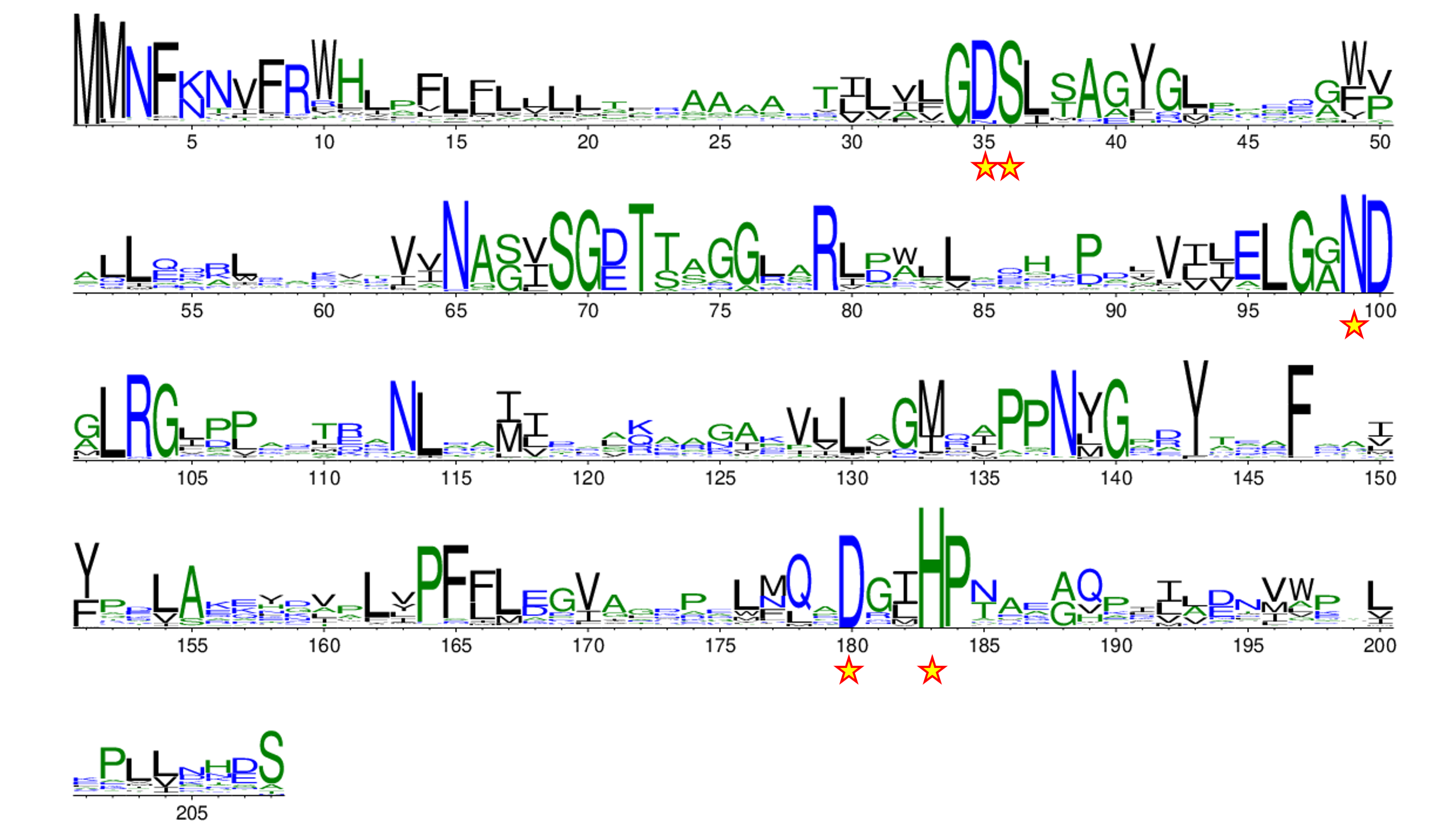

Supplement: S6 Fig — Conservation WebLogo was constructed from a multiple sequence alignment of 12,172 TesA homologs. Stars indicate the positions of key residues within the active site that were tested by mutagenesis (Asp35, Ser36, Asn99, Asp180, and His183). (TIFF) [file pbio.3003427.s009.tiff]

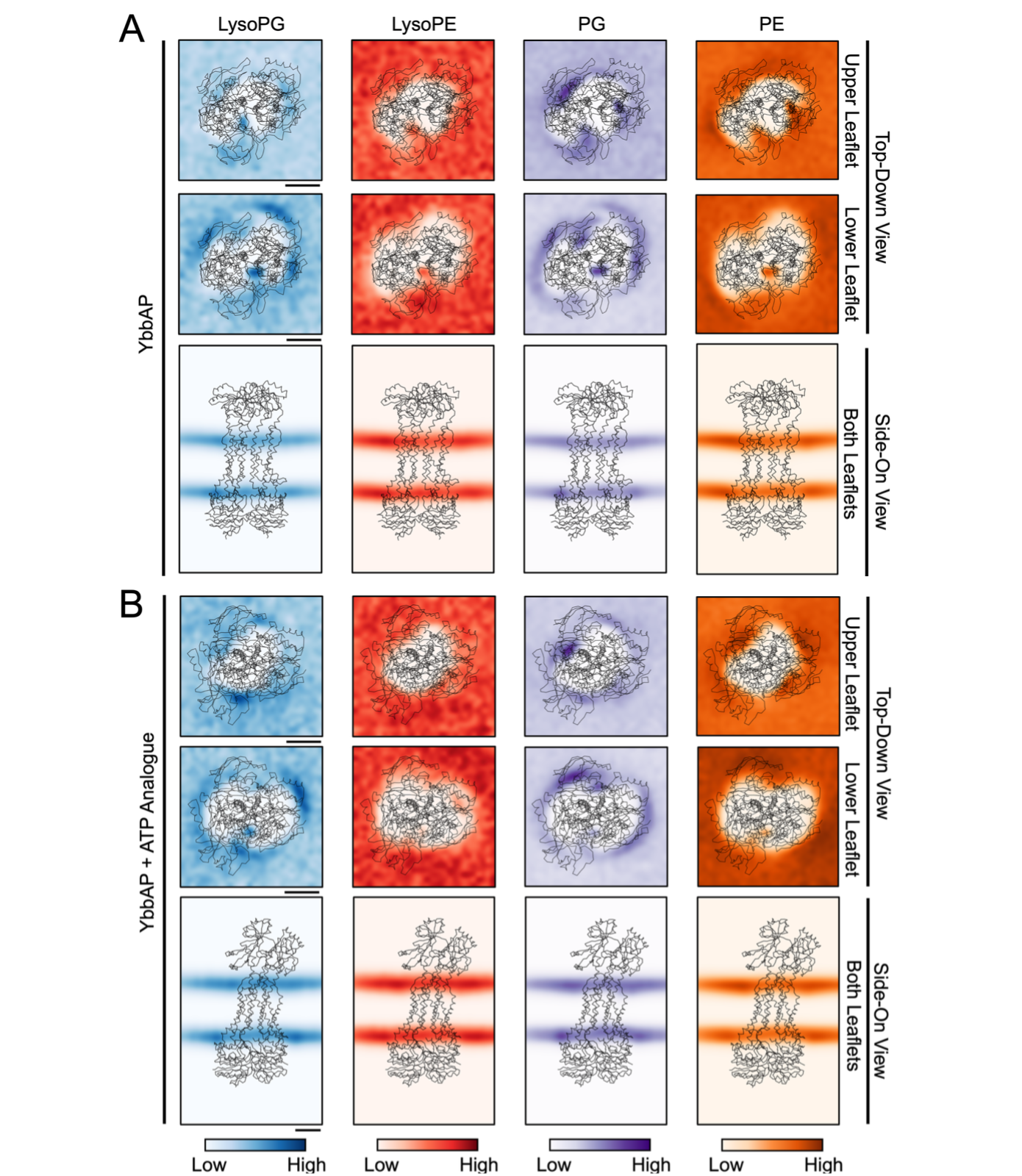

Supplement: S7 Fig — 2D histograms (bins: 30 × 30) of frequency density of lipid headgroup position (LysoPG—blue, LysoPE—red, PG—purple, PE—orange) in the membrane over 15 μs coarse-grained simulation repeats of YbbAP in the (A) apo (n = 3) and (B) nucleotide-bound state (n = 3), normalized to the maximum residency per plot. Calculated from a trajectory of frames comprising of every 10th 1 ns simulation time point. Protein backbone coordinates are shown in black. Each scale bar represents 20 Å. (TIFF) [file pbio.3003427.s010.tiff]

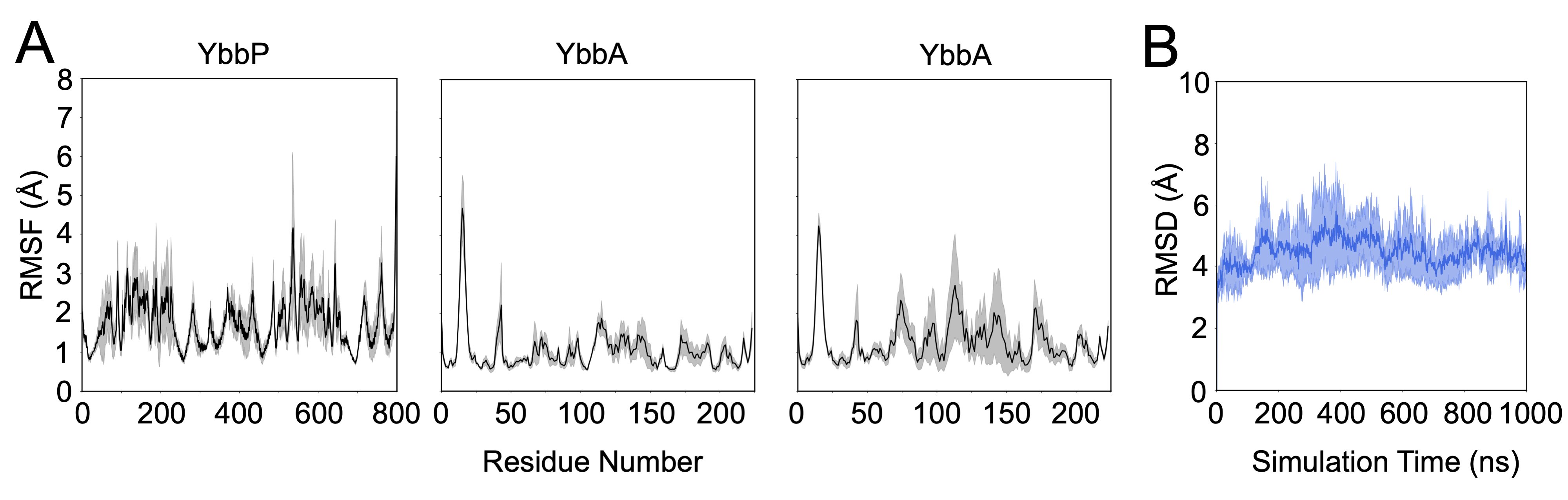

Supplement: S8 Fig — (A) RMSF per residue over 1,000 ns of atomistic simulation repeats (n = 3). Mean RMSF value per residue is shown in black, with mean ± 1 standard deviation shown in gray. (B) RMSD of YbbAP backbone (in comparison to the start frame) over 1,000 ns of atomistic simulation repeats (n = 3). Mean RMSF value per residue is shown in blue, mean ± 1 standard deviation shown in light blue. Underlying data can be found in S14 Data. (TIFF) [file pbio.3003427.s011.tiff]

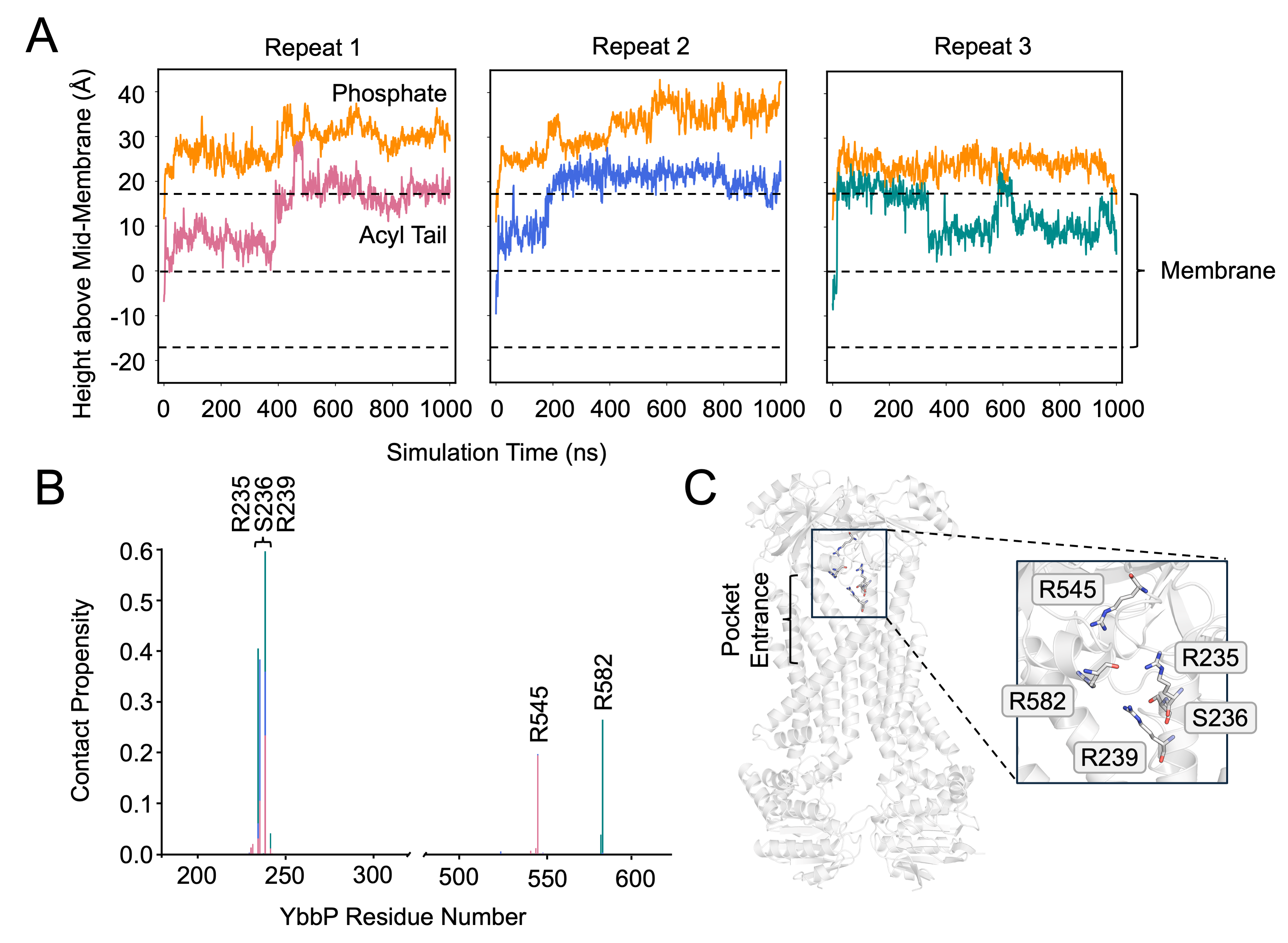

Supplement: S9 Fig — (A) Vertical movement of the LysoPG molecule that begins each atomistic simulation repeat within the periplasmic pocket. Repeats 1 (pink) and 2 (blue) show initial extraction of the phosphate head group, followed by the lipid tail over the course of the simulation. Repeat 3 (teal) shows partial extraction of LysoPG from the membrane, which is reversed over the course of the simulation. (B) Contact propensity between the LysoPG and YbbP. Contact Propensity is defined as the fraction of frames where LysoPG is within 4 Å of a given residue. The bar chart shows stacks of values over three repeats. (C) Key residues lining the periplasmic pocket identified during lipid contact analysis that may form interactions with LysoPG during extraction from the bilayer. The atomistic simulation is shown as S4 Movie. Underlying data can be found in S15 Data. (TIFF) [file pbio.3003427.s012.tiff]

## Slide 1
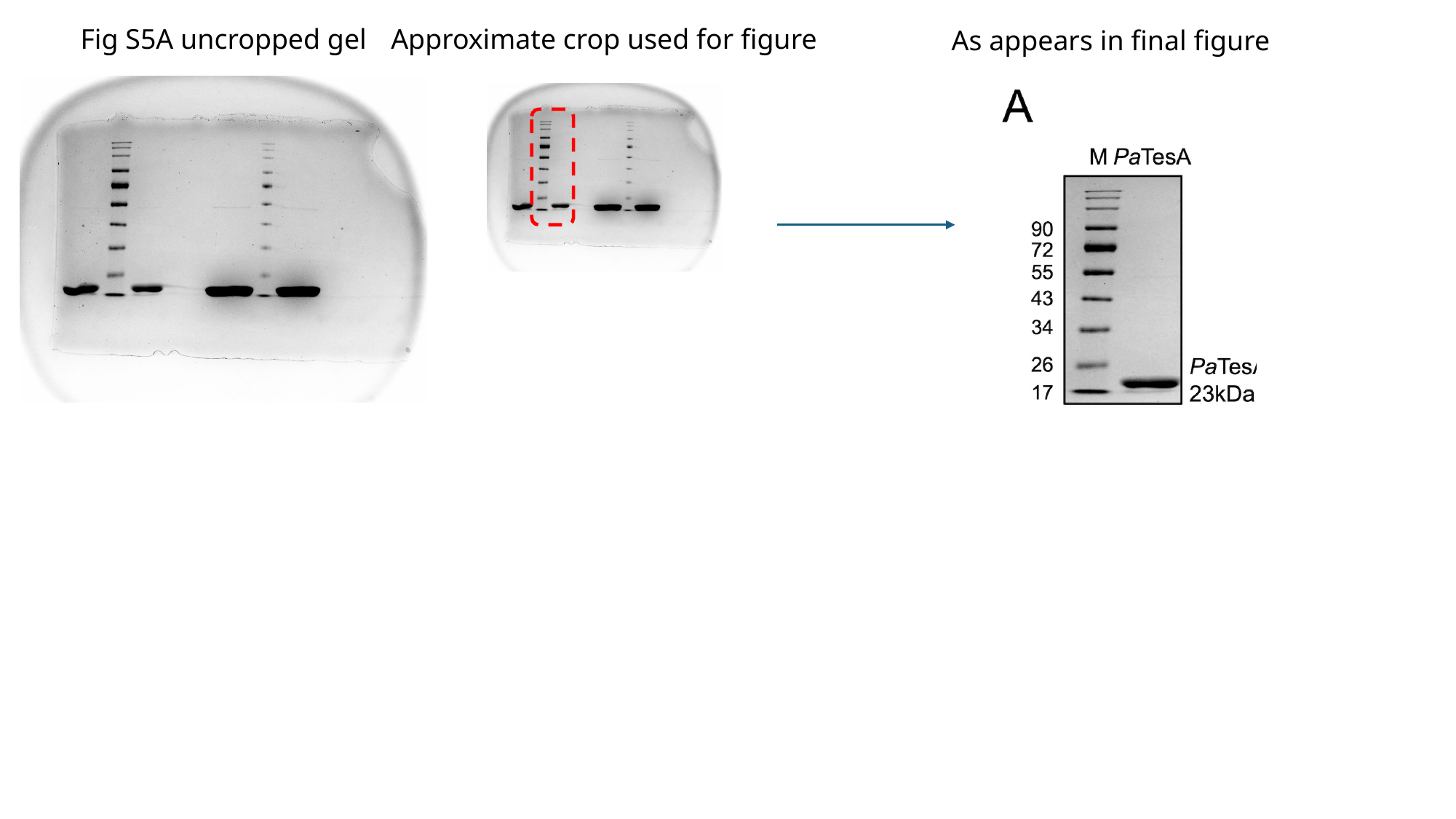

Fig S5A uncropped gel
Approximate crop used for figure
As appears in final figure

Supplement: S11 Data — (PPTX) [file pbio.3003427.s028.pptx]
