# Supplementary material for: Structural characterization of the YbbAP-TesA ABC transporter identifies it as a lipid hydrolase complex that extracts hydrophobic compounds from the bacterial inner membrane
Source: PLoS Biol. 2025 Nov 25;23(11):e3003427. doi: 10.1371/journal.pbio.3003427 (PMC12646458; doi:10.1371/journal.pbio.3003427)
Supplement: S3 Data — (PPTX) [file pbio.3003427.s020.pptx]

## Slide 1
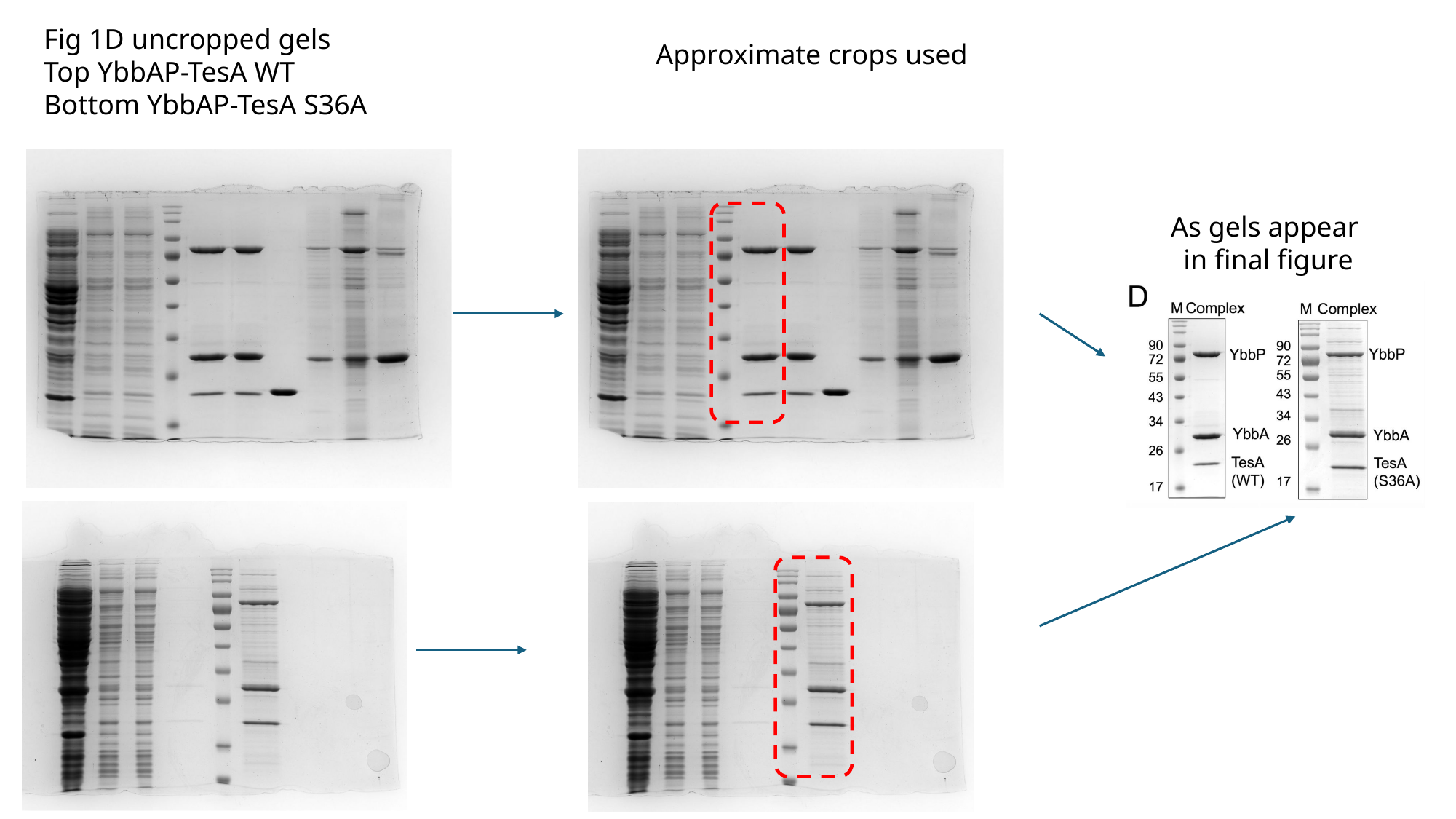

Fig 1D uncropped gels
Top YbbAP-TesA WT
Bottom YbbAP-TesA S36A
Approximate crops used
As gels appear
 in final figure
